# Modulation of the Tomato Rhizosphere Microbiome via Changes in Root Exudation Mediated by the Ethylene Receptor NR

**DOI:** 10.3390/microorganisms9122456

**Published:** 2021-11-28

**Authors:** Ruixin Fu, Haichao Feng, Francisco Dini-Andreote, Zhen Wang, Chunbin Bo, Wenhui Cao, Keming Yang, Mingchun Liu, Tianjie Yang, Qirong Shen, Yangchun Xu, Zhong Wei

**Affiliations:** 1 Jiangsu Provincial Key Lab of Solid Organic Waste Utilization, Jiangsu Collaborative Innovation Center of Solid Organic Waste Resource Utilization, National Engineering Research Center for Organic-Based Fertilizers, Nanjing Agricultural University, Nanjing 210095, China; 2016203021@njau.edu.cn (R.F.); hcfeng@njau.edu.cn (H.F.); wz18851000927@163.com (Z.W.); 2020103124@stu.njau.edu.cn (C.B.); cwh1808123@163.com (W.C.); 2017203022@njau.edu.cn (K.Y.); shenqirong@njau.edu.cn (Q.S.); ycxu@njau.edu.cn (Y.X.); weizhong@njau.edu.cn (Z.W.); 2 Department of Plant Science and Huck Institutes of the Life Sciences, The Pennsylvania State University, University Park, PA 16801, USA; fjd5141@psu.edu; 3 Key Laboratory of Bio-Resource and Eco-Environment of Ministry of Education, College of Life Sciences, Sichuan University, Chengdu 610065, China; mcliu@scu.edu.cn

**Keywords:** ethylene, never-ripe (*Nr*), rhizosphere, microbiome, root exudation

## Abstract

Plant hormones have been recently shown to exert an indirect influence on the recruitment of plant-associated microbiomes. However, it remains unclear the extent to which the disruption of the ethylene (ET) signaling pathway affects the assembly and functioning of plant-root microbiomes. In this study, the Never-ripe tomato mutant (*Nr*) was profiled for differences compared to the wild type (control). Tomato plants were subjected to root exudate profiling and the characterization of bacterial and fungal communities. Compared to the control, *Nr* revealed differences in the composition of root exudates, including lower amounts of esculetin, gallic acid, L-fucose, eicosapentaenoic acid, and higher amounts of β-aldehyde. Interestingly, *Nr* significantly differed in the composition and functioning of the rhizosphere bacterial community. We also identified the taxa that occurred at relatively higher abundances in *Nr*, including the genus *Lysobacter*, which displayed a significant negative correlation with changes in eicosapentaenoic acid and esculetin, and a significant positive correlation with changes in β-aldehyde. Taken together, our study provides evidence that a mutation in the ET receptor exerts predictable changes in the root-associated microbial taxa of tomato plants. These indirect effects can potentially be explored towards new strategies to engineer beneficial plant microbiomes via targeted changes in plant genetics and physiology.

## 1. Introduction

Plants growing in soil are dynamically interacting with microbes, including bacteria, fungi, protists, and viruses [1,2]. These distinct associations expand the genomic and functional capabilities of plants to explore the environment and develop resistance to biotic and abiotic stresses, also termed as “the plant’s second genome” [3]. The plant-root microbiomes contribute to plant fitness by enhancing water and nutrient uptake [4], promoting resistance/tolerance to abiotic stresses [5], protecting against pathogens [3], and reducing herbivory by priming plant defenses [6]. Thus, understanding the factors and mechanisms controlling the establishment of beneficial plant microbiomes has recently gained attention in the literature [2,7,8,9].

Plant-root microbiomes are modulated by several biotic and abiotic factors that vary in importance across distinct scenarios. For instance, plant genotype [10,11,12] and growth stage [13,14] can exert significant influences on root-microbiome composition. It was previously shown that three *bx* (Benzoxazinoids) mutants (i.e., with disruption in different steps in the BX pathway) had a significant effect in selecting distinct rhizosphere bacterial communities, albeit fungal communities were found to be similar across mutant lines [15]. In addition, the root microbiome of *Arabidopsis thaliana* at the seedling stage was significantly different from those found at later stages of plant development (flowering/senescence) [16]. Taken together, these differences are often attributed to variations in root traits and exudation [17]. In addition, it has been demonstrated that plant hormones can also exert an influence on the recruitment and establishment of plant microbiomes [9,18]. For example, changes in the jasmonic acid (JA) pathway and its derivatives were shown to affect the relative abundances of specific microbial taxa in association with *A. thaliana* [19]. Similarly, it was found that the ethylene (ET) pathway indirectly affects the recruitment of *Catenulispora* sp. in the rhizosphere of peanuts [20]. Plant hormones (e.g., salicylic acid, jasmonic acid, and ethylene) can interactively regulate plant growth and health [21,22]. In doing so, these physiological changes indirectly modify the selection and establishment of root microbiome taxa, thus affecting the set functions they provide.

Ethylene is involved in a wide range of developmental processes and physiological responses, such as plant ripening and responses to biotic stresses [23,24,25]. Ethylene exerts various physiological functions involved in the perception and signaling transduction pathways [22,26]. The ET receptors are encoded by a multigene family of proteins similar to the bacterial two-component histidine kinases. The *Le-ETR3* (*Never ripe*, *Nr*) is one member of the multigene family consisting of at least five members present in tomato plants [27]. The tomato *Nr* mutant has a disruption in one of the ET receptors ETR3 (NR), which confers ET insensitivity and produces non-ripening fruits [28]. Previous studies showed the role of exogenous ET in structuring the peanut rhizosphere microbiome [20]. However, it still remains to be addressed the extent to which ET regulated by *NR* affects the rhizosphere microbiomes via inducible changes in plant-root exudates.

Here, we used the wild-type tomato (*Solanum lycopersicon* cv. Micro-Tom, hereafter “WT”) and an isogenic *NR* mutant line (hereafter “*Nr*”) as model plants to study the influence of ET on induced changes in root exudates and their impact on the rhizosphere microbiome. In this study, we characterized the root exudates and rhizosphere microbiome (bacterial and fungal communities) of WT and *Nr* plants at the seeding, flowering, and fruiting stage. We hypothesized that *Nr* plants would differ from WT in both bacterial and fungal community compositions as a result of chemical differences in root exudates. Furthermore, we expected these differences in microbiomes to become greater at the later stages of plant development. This would occur as a result of initial differences in microbial taxa recruitment that intensify as the root microbiome develops over time. Last, we expect the initial bacterial communities to be more variable than the fungal communities when comparing *Nr* and WT. In addition, we expect these differences in the bacterial community composition to be correlated with specific root exudates in these plants across different developmental stages.

## 2. Materials and Methods

### 2.1. Plant Material and Growth

The wild type (WT) and *NR* mutant (*Nr*) tomato (*Solanum lycopersicon* cv. ‘Micro-Tom’) seeds used in this study were provided by Mingchun Liu [29] at the Sichuan University. The seeds were surface-sterilized, as similarly performed in Wei et al. [30]. First, seeds were drenched using 3% NaClO for 5 min and in 70% ethanol for 1 min. After that, seeds were washed with sterile water at least five times and germinated on water-agar plates in the dark for three days at 30 °C. Seedlings were then sown into seedling plates containing 200 g of the seedling substrate (Huainong, Huaian Soil and Fertilizer Institute, Huaian, China). Tomato plants were placed in a controlled glass greenhouse (25 °C to 30 °C, 70% relative humidity, 16 h light/8 h dark photoperiod, 200 µmoL m^−2^ s^−1^ PPFD). Tomato plants were transplanted at the three-leaf stage (that is, 17 days after sowing) for the following experiment.

### 2.2. Experimental Soils

Field soil was collected from a healthy tomato field in Qilin town (118°57′ E, 32°03′ N), Nanjing, China. The soil used in this study was classified as Udic Argosol, containing 24.6 g kg^−1^ of total organic matter, 6.3 g kg^−1^ of total N, 172.9 mg kg^−1^ of available *P*, 178 mg kg^−1^ of K, pH of 5.4, soil electric conductivity (EC) of 0.278 ms cm^−1^. After removing surface debris on the ground, the soil was collected from a depth of 5 to 20 cm. All experimental soils were sieved (<4 mm) and homogenized thoroughly for the following experiment.

### 2.3. Collection of Root Exudates

In order to explore the effects of *NR* on root exudation profile, we collected root exudates from WT and *Nr* plants grown in axenic conditions at three stages (seedling, flowering, and fruiting). To date, many approaches have been used to collect root exudates, but each has its advantages and shortcomings [31]. The use of hydroponics minimizes the unevenness of dispersal of root exudates in the collecting system. Besides, hydroponics prevents potential noise due to chemical co-metabolisms and absorption by soil particles, nutrients, and the soil biota [19,32]. Despite the possibility that root morphology may change in the liquid solution, the confounding effects caused by microbes in solid matrixes (e.g., soil) can significantly hinder the proper detection of root exudate composition. Therefore, we considered the advantages of using the nutrient solution in a sterile system to cultivate plants for root exudates collection. This strategy nicely aligns with the pre-defined research goal (‘evaluation of the effects of NR on root exudate profile under controlled conditions’) [17,31]. In detail, seeds of WT and *Nr* plants were surface-sterilized and germinated as mentioned above. Germinated seeds were first washed three times with sterile water and transferred into sterile tissue culture bottles containing sterilized vermiculite (Xianlin Garden Center, Nanjing) for growth. After incubation for 14 days in a controlled greenhouse (25 °C to 30 °C, 70 % relative humidity, with a photoperiod of 16 h light/8 h dark, 200 µmoL m^−2^ s^−1^ PPFD), seedlings were transplanted into 50 mL conical flasks containing 50 mL sterile liquid 1/4 sucrose-free Murashige Skoog (MS) medium [33]. As tomato plants grow taller during the experiment, unsampled tomato plants at the flowering and fruiting stage were later transferred to 100 mL sterile conical flasks with 80 mL 1/4 MS medium. The MS medium was changed every two days during the growth period to minimize potential microbial growth.

Before root exudates were collected, each conical flask was checked for sterility by plating 100 μL aliquots onto Luria-Bertani (LB) agar medium (peptone 10 g L^−1^, yeast extract 5 g L^−1^, NaCl 5 g L^−1^, agar 15 g L^−1^). Plants at the seedling, flowering, and fruiting stages (i.e., 14, 37, and 47 days after transferring to the conical flasks, respectively) were sampled from the flasks containing 1/4 MS medium. The tomato roots were washed three times with sterile distilled water and transferred into new sterile conical flasks containing fresh sterile distilled water. These plants were maintained in the system for 48 h before root exudate collection. Root exudate aliquots were filtered through 0.45 μm Millipore filters to remove particles and other contaminants. All the exudate solution was then lyophilized and stored at −80 °C. In total, we obtained 18 root exudate samples (two genotypes × three replicates × three stages).

### 2.4. LC-MS/MS Analysis, Data Preprocessing and Annotation of Root Exudates

The collected root exudates from three individual biological replicates of each tomato line at three different growth stages were analyzed using a UHPLC–QTOF–MS at Shanghai BIOTREE biotechnology limited company, China. In brief, LC-MS/MS analyses were performed using a UHPLC system (1290, Agilent Technologies Inc., Santa Clara, CA, USA) with a UPLC BEH Amide column (1.7 μm, 2.1 × 100 mm, Waters, Milford, MA, USA) coupled with a Triple TOF 5600 (Q-TOF, AB Sciex, Foster City, CA, USA). The mobile phase consisted of 25 mM NH_4_OAc and 25 mM NH_4_OH in water (pH = 9.75) (A) and acetonitrile (B). These were carried out with an elution gradient as follows: 0 min, 95% B; 7 min, 65% B; 9 min, 40% B; 9.1 min, 95% B; 12 min, 95% B, which was delivered at 0.5 mL min^−1^; using an injection volume of 2 μL. The Triple TOF mass spectrometer was used for its ability to acquire MS/MS spectra on an information-dependent acquisition (IDA) during an LC/MS experiment. In this mode, the acquisition software (Analyst TF 1.7, AB Sciex, Foster City, CA, USA) continuously evaluates the full scan survey MS data as it collects and triggers the acquisition of MS/MS spectra depending on the preselected criteria. In each cycle, 12 precursor ions with intensities greater than 100 were chosen for fragmentation at the collision energy (CE) of 30 V (15 MS/MS events with product ion accumulation time of 50 msec each). ESI source conditions were set as follows: ion source gas 1 as 60 Psi, ion source gas 2 as 60 Psi, curtain gas as 35 Psi, source temperature 650 °C, ion spray voltage floating (ISVF) 5000 V or −4000 V in positive or negative modes, respectively. For the identification of metabolites, all the authentic standards in the database have already been tested to obtain the specific retention times (RT) and mass-to-charge ratios (*m*/*z*) by using both HPLC and mass spectrometry, respectively. All of the peaks detected in the root exudate samples were then compared with the database to infer the identity of the peaks based on the database information (‘authentic standards’).

Raw data files of mass spectrometry (.wiff) were converted to the mzXML format using ProteoWizard, and processed using the R package XCMS (version 3.2) [34]. The pre-processing results generated a data matrix that consisted of the retention time (RT), mass-to-charge ratio values (*m*/*z*), and peak intensity. The R package *CAMERA* (version 1.40.0) [35] was used for peak annotation after XCMS data processing. A second mass spectrometer (MS2) database was built using HMDB (version 4.0), MONA (version 3, https://mona.fiehnlab.ucdavis.edu/, 18 June 2020), and the Metlin databases combined with an in-house MS2 database.

### 2.5. Continuous and Nondestructive Sampling for Rhizosphere Soils of Tomato Plants

We used the ‘rhizobox’ method [30] filled with natural soil (aforementioned in the experimental soils section), with a slight modification to repeatedly collect rhizosphere soils from each individual plant of WT and *Nr* lines without damaging the root system. The rhizobox system (Figure S1) consisted of three-layer cylinders (110 × 80 mm, height × diameter). The interior layer had a 50 μm nylon mesh net to contain the root growth, the intermediate layer had ten combined nylon bags of 150 μm (ca., 8–21 × 120 mm, width × length), and the exterior layer consisted of a 4 mm metal net. Each rhizobox system was filled with 3.4 g of homogenized field soil, and the intermediate layers were sampled individually throughout the experiment. Tomato plants were placed in controlled greenhouse (25 °C–30 °C, 70% relative humidity, 16 h light/8 h dark photoperiod, 200 µmol m^−2^ s^−1^ PPFD). We continuously sampled the rhizosphere soil from these plants at the seedling (14 days after transplantation), flowering (37 days after transplantation), and fruiting (47 days after transplantation) developmental stages. Six biological replicates were sampled per treatment at each stage. Each replicate consisted of one composite sample obtained from four individual plants. In total, we obtained 36 rhizosphere soil samples (two genotypes × six replicates × three stages). Rhizosphere soil samples were stored at −20 °C for further processing.

### 2.6. DNA Extraction and Metagenome Sequencing

A total of 0.5 g of rhizosphere soil was used as initial material to extract the total DNA using the PowerSoil DNA Isolation Kit (MoBio, Carlsbad, CA, USA), following the manufacturer’s protocols. For each sample, 1 μg of genomic DNA was sheared using a Covaris S220 Focused-ultrasonicator (Woburn, MA USA), and the sequencing libraries were prepared with a fragment length of approximately 450 bp. All samples were sequenced in the Illumina HiSeq X instrument with paired-end 150 bp (PE150) mode at the Shanghai Biozeron BioTech. Co. Ltd. The output raw data was ca. 10 Gb per sample.

Raw sequence reads underwent quality trimming using Trimmomatic (http://www.usadellab.org/cms/uploads/supplementary/Trimmomatic, 18 June 2020) to remove adaptor contaminants and low quality reads [36]. In total, we acquired clean reads ranging from 54,657,284 to 80,460,250 per sample (Table S1).

Clean sequence reads were used to obtain a set of contigs in each sample using MegaHit with the “--min-contig-len 500” parameters [37]. Open reading frames (ORFs) of assembled contigs were predicted using Prodigal (v2.6.3) [38], and all ORFs were clustered based on sequence identity to generate a unique set using CD-HIT (parameters: -n 9 -c 0.95 -G 0 -M 0 -d 0 -aS 0.9 -r 1) [39]. The longest sequence in each cluster was considered as the representative sequence of each gene in the unique-gene dataset.

We translated the unique-gene dataset into putative amino acid sequences and aligned them against the non-redundant NCBI nr (download 20 May 2020) database, and annotated against the KEGG database (http://www.genome.jp/kegg/, May 26th, 2020, KO without animal and plant sequences) using blastp (version 2.2.31+) with e-value < 1 × 10^−5^. The taxonomic information (domain, kingdom, phylum, class, order, family, genus, and species) of each gene was obtained using MEGAN based on the LCA (lowest common ancestor) method [40]. We used the tool BlastKOALA to functionally annotate the unique gene in the KEGG database (release 86.1) [41]. In total, we obtained unique 68,826 nr taxa and 73,010 KEGG annotations (Table S1).

In order to calculate the gene abundance within total samples, we used the salmon software [42] to obtain the total number of reads for each gene. The gene abundance was calculated as follows:

Ab(S)=Ab(U)+Ab(M)

$$Ab\left(U\right)=\overset{M}{\underset{i=1}{\sum }}1/l$$

$$Ab\left(M\right)=\overset{M}{\underset{i=1}{\sum }}\left(Co*1\right)/l$$

$$Co=\frac{Ab\left(U\right)}{\sum_{i=1}^{N}Ab\left(U_{i}\right)}$$

*Ab*(*S*), gene abundance; *Ab*(*U*), single-mapping reads abundance; *Ab*(*M*), multi-mapping reads abundance; *l*, length of gene sequence [43].

### 2.7. Statistical Analyses

Principal component analysis (PCA) and PERMANOVA (based on Bray-Curtis, permutation = 999) [44,45] was performed based on the abundance data of metabolic ions to evaluate differences in root exudates between plant lines and across developmental stages. These analyses were carried out using the R package vegan [46] based on the Hellinger-transformed data. The detection of discriminating root exudate metabolites between WT and *Nr* samples were obtained using the DESeq2 generalized linear models [47].

Taxonomic information for bacteria and fungi was obtained based on the NCBI nr database at the species level. Alpha diversity (Shannon index and species richness) was calculated using the R package vegan [46] based on Hellinger-transformed data. Beta diversity of bacterial and fungal communities were analyzed and visualized using PCA (R package vegan) based on the Hellinger transformation for data normalization. To test for statistical differences in bacterial taxa based on the metagenomes from each treatment, we used STAMP (v2.1.3, Canada) [48]. Venn diagrams were constructed to visualize the proportion of groups exclusive and shared between samples using a web source (http://bioinformatics.psb.ugent.be/webtools/Venn/, 18 March 2021). Functional KEGG annotations were based on the KEGG database. The functional dissimilarities of bacterial communities between WT and *Nr* plants were analyzed and visualized using PCA in the R package vegan. Differences in bacterial taxonomic and functional annotations across treatments were tested using Permutational multivariate analysis of variance (PERMANOVA) (Adonis, transformed data by Bray-Curtis, permutation = 999) [44,45]. Last, we used STAMP to analyze differences in the KEGG level 2 annotation pathways between WT and *Nr* plants.

Multiple correlation analysis between *Nr*-related bacterial taxa (at the species level by NCBI nr database annotation) obtained from STAMP and *Nr*-related metabolites obtained from the DESeq2 generalized linear models was conducted by *Pearson* correlation. Since root exudate metabolome and microbiome analyses were not performed at the same sample, it was not possible to compare these directly. Accordingly, the three samples from the six metagenome replicates were randomly chosen, and then metagenomic sequencing and root exudates datasets were randomly paired at random 10,000 times and the average *Pearson* correlation was calculated between the variance values. To reduce the number of calculations, we only showed significant differences of microbial taxa (relative abundances, RA that were >0.01 %) and root exudate metabolites detected at the three different growth stages between the WT and *Nr* plants. When comparing the mean differences between two treatments, we used analysis of Student’s *t*-tests and Welch’s *t*-test, where *p*-values below 0.05 were considered as statistically significant. All data were analyzed to compare differences between the treatments with SigmaPlot 14.0, STAMP [48], and R v.3.5.1 [49].

## 3. Results

### 3.1. Chemical Characterization of WT and Nr Plants Exudates at Distinct Stages of Plant Development

**Figure 1:**
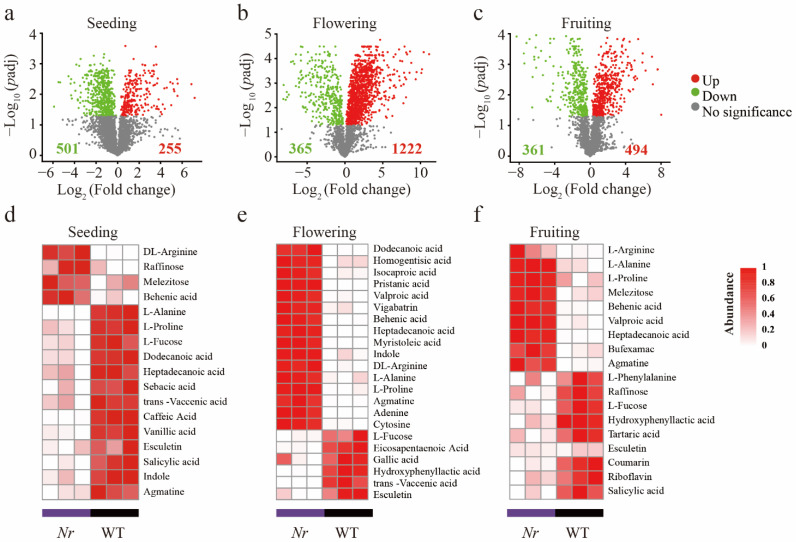
The effect of *NR* on root exudation profiles of WT and *Nr* tomato lines. Volcano plots show the statistical significance (y-axis) against fold-change (x-axis) of all ions considered in the root exudates. Red, green, and gray symbols indicate significantly increased, decreased, and un-related ions in abundance (*p*-adjusted < 0.05) at the seedling (**a**), flowering (**b**), and fruiting (**c**) stage, respectively. Heatmap shows the abundance of metabolites in root exudates that are significantly influenced by NR at the seedling (**d**), flowering (**e**), and fruiting (**f**) stage, respectively.

We identified a total of 2707 ions across all samples using UHPLC–QTOF–MS (1266 ESI^−^ and 1440 ESI^+^). Based on metabolic ions relative abundance, PCA and Two-way PERMANOVA (based on 999 permutations) showed that both plant developmental stage and Nr significantly explained the observed variation in tomato root exudates (Figure S2a and Table S2). We found that exudates of *Nr* plants had significantly higher relative abundances of fatty acids and plant hormones than that of WT at the three different developmental stages (Figure S2b–d). However, the concentrations of alcohols, amines, sugars, organic acids, and nucleic acids in *Nr* plants were significantly lower than those found in the WT at the fruiting stage (Figure S2d). We then focused on root exudate metabolic ions that significantly changed in concentration in *Nr* plant compared to WT using DESeq2. In general, compared to the WT, among the metabolic ions affected by *NR*, 8.31%, 45.14%, and 18.25% significantly increased in concentration, and 18.51%, 13.48%, and 13.34% significantly decreased at the seedling, flowering, and fruiting stage, respectively (Figure 1a–c, Table S3 and Supplementary File S1). Specifically, compared to the WT, DL-arginine, L-alanine, L-proline, raffinose, melezitose, behenic acid, and agmatine were at significantly higher concentrations in *Nr* plants in at least two growth stages (Figure 1d–f). Likewise, L-fucose, salicylic acid, esculetin, and hydroxyphenyl acetic acid were at significantly lower concentrations in *Nr* plants in at least two growth stages (Figure 1d–f). It is worth mentioning that in the *Nr* plants, a significant decrease in the concentration of coumarin was detected at the fruiting stage (Figure 1f).

### 3.2. Profiling the Root Microbiome of WT and Nr at Distinct Stages of Plant Development

**Figure 2:**
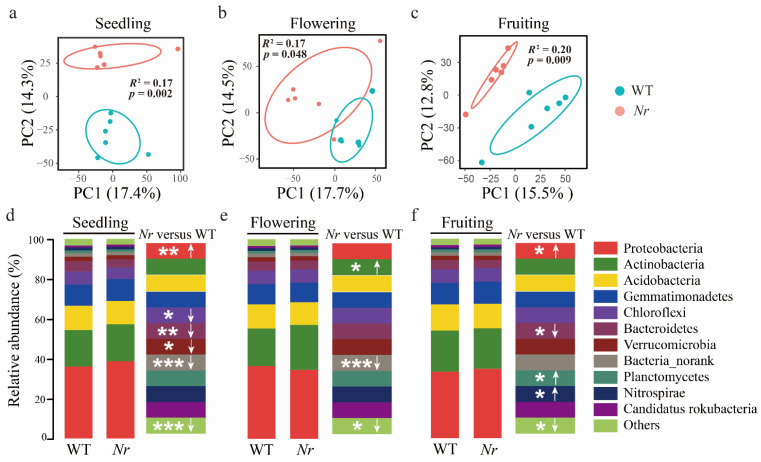
Bacterial community structure in the rhizosphere of WT and *Nr* tomato plants. Principal component analysis (PCA) of the rhizosphere bacterial communities of WT and *Nr* plants at the seedling (**a**), flowering (**b**), and fruiting (**c**) stages. Bar chart displaying the relative abundances of bacterial phyla in the rhizosphere of WT and *Nr* plants at the seedling (**d**), flowering (**e**), and fruiting (**f**) stages. Two-sided Student’s *t*-test (upward and downward arrows indicate significant increases and decreases in taxa relative abundances, respectively), * indicates *p* < 0.05, ** indicates *p* < 0.01 and *** indicates *p* < 0.001.

We analyzed the patterns of bacterial and fungal alpha diversity, and obtained taxonomic information using the NCBI nr database at the species level. Overall, we found no significant differences for the Shannon index and species richness for bacterial (Figure S3a,c and Table S4) or fungal (Figure S3b,d and Table S4) communities when WT and *Nr* plants were compared across stages. PERMANOVA analysis revealed only bacterial communities—and not fungal communities—to significantly differ in terms of beta-diversity between WT and *Nr* across all developmental stages (Figure S4a–c). Moving forward, a focus was set on bacterial rather than fungal community differences across these treatments. Two-way PERMANOVA analysis revealed that both plant developmental stage, *Nr,* and their interactions had significant effects on the structure of the rhizosphere bacterial communities (Tables S5 and S6). We found the bacterial community structure in the rhizosphere of WT and *Nr* plants to be significantly different at the seedling (PERMANOVA: *F*_1,10_ = 2.04, *R*^2^ = 0.1696, *p* = 0.002, Figure 2a), flowering (PERMANOVA: *F*_1,10_ = 2.09, *R*^2^ = 0.17, *p* = 0.048, Figure 2b) and fruiting stages (PERMANOVA: *F*_1,10_ = 2.42, *R*^2^ = 0.20, *p* = 0.009, Figure 2c). Moreover, these differences became greater at later stages of plant development (Figure 2a–c).

Our results indicated that the dominant bacterial phyla in the rhizosphere of WT tomato plants were Proteobacteria (35.67 ± 2.20%), Actinobacteria (19.84 ± 2.22%), Acidobacteria (12.12 ± 0.83 %), and Gemmatimonadetes (10.56 ± 0.94%) at the phylum level, which were annotated with NCBI nr database. The relative abundances of some bacterial phyla showed significant differences between WT and *Nr* plants at the three growth stages (Figure 2d–f). For instance, the relative abundance of Proteobacteria in *Nr* plants was significantly higher at the seedling stage (*p* = 0.006, *t* = 3.52, df = 10, Figure 2d) and the fruiting stage (*p* = 0.047, *t* = 2.27, df = 10, Figure 2f). Conversely, the relative abundance of Actinobacteria was significantly higher at the flowering stage (*p* = 0.035, *t* = 2.44, df = 10, Figure 2e) in *Nr* compared to WT. In order to compare the differentially abundant bacteria between WT and *Nr* at the species level (annotated with NCBI nr database), we used STAMP analysis to identify potential differences in bacterial taxa relative abundances between WT and *Nr*. The result indicates that the *Nr*-differentially abundant bacterial taxa represented 9.55%, 10.18%, and 8.96% of the entire communities at the seedling, flowering, and fruiting stages, respectively (Table S7 and Supplementary File S2). Moreover, a total of 343, 317, and 411 unique bacterial taxa were found in *Nr* plants at the seedling, flowering, and fruiting stages, respectively (Figure S5a–c).

**Figure 3:**
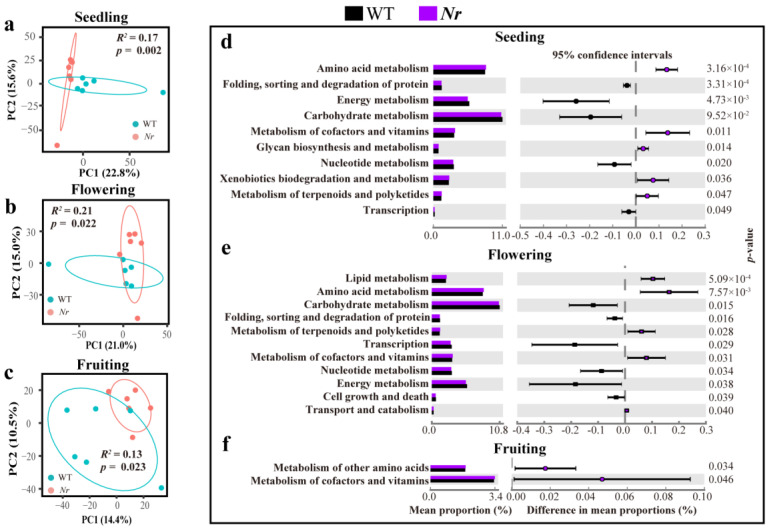
The functional potential of bacterial communities in the rhizosphere of WT and *Nr* plants. Principal component analysis (PCA) based on functional annotations of the rhizosphere bacterial community of WT and *Nr* plants at the seedling (**a**), flowering (**b**), and fruiting (**c**) stages. The functional KEGG level 2 pathway annotation was used for the comparison between WT and *Nr* plants at the seedling (**d**), flowering (**e**), and fruiting (**f**) stages. *p*-values are shown as inset panels based on two-sided Welch’s *t*-test.

Next, the genetic potential of bacterial communities was analyzed and visualized using PCA combined with PERMANOVA, based on KEGG functional annotation. Our results showed the functional profiles of the rhizosphere bacterial communities between WT and *Nr* lines to be significantly different at the seedling (PERMANOVA: *F*_1,10_ = 2.00, *R*^2^ = 0.17, *p* = 0.002, Figure 3a), flowering (PERMANOVA: *F*_1,8_ = 2.16, *R*^2^ = 0.21, *p* = 0.022, Figure 3b) and fruiting stages (PERMANOVA: *F*_1,10_ = 1.45, *R*^2^ = 0.13, *p* = 0.023, Figure 3c). However, we found no significant difference between the overall functional profiles of the fungal community between lines or across developmental stages (Figure S6a–c). Based on KEGG pathway annotation at level 2, STAMP analysis of bacterial communities revealed that *Nr* plants had significantly higher relative abundances of amino acid metabolism pathway and metabolism of cofactors and vitamins at the three stages (Figure 3d–f), and functions related to the metabolism of terpenoids and polyketides of *Nr* plants showed significantly higher relative abundances than that of WT, but only at the two initial stages (Figure 2d,e).

### 3.3. Correlations between Nr-related Bacterial Taxa and Root Exudate Metabolites

We tested for potential correlations between WT and *Nr* differentially abundant bacterial taxa and root-specific metabolites. This included four types of associations, as follows. Type *i*: taxa at a higher relative abundance in WT and positively correlated with root exudate metabolites. Type *ii*: taxa at a higher relative abundance in WT and negatively correlated with root exudate metabolites. Type *iii*: taxa at a higher relative abundance in *Nr* plants and positively correlated with root exudate metabolites. Type *iv*: taxa at a higher relative abundance in *Nr* plants and negatively correlated with root exudate metabolites.

**Figure 4:**
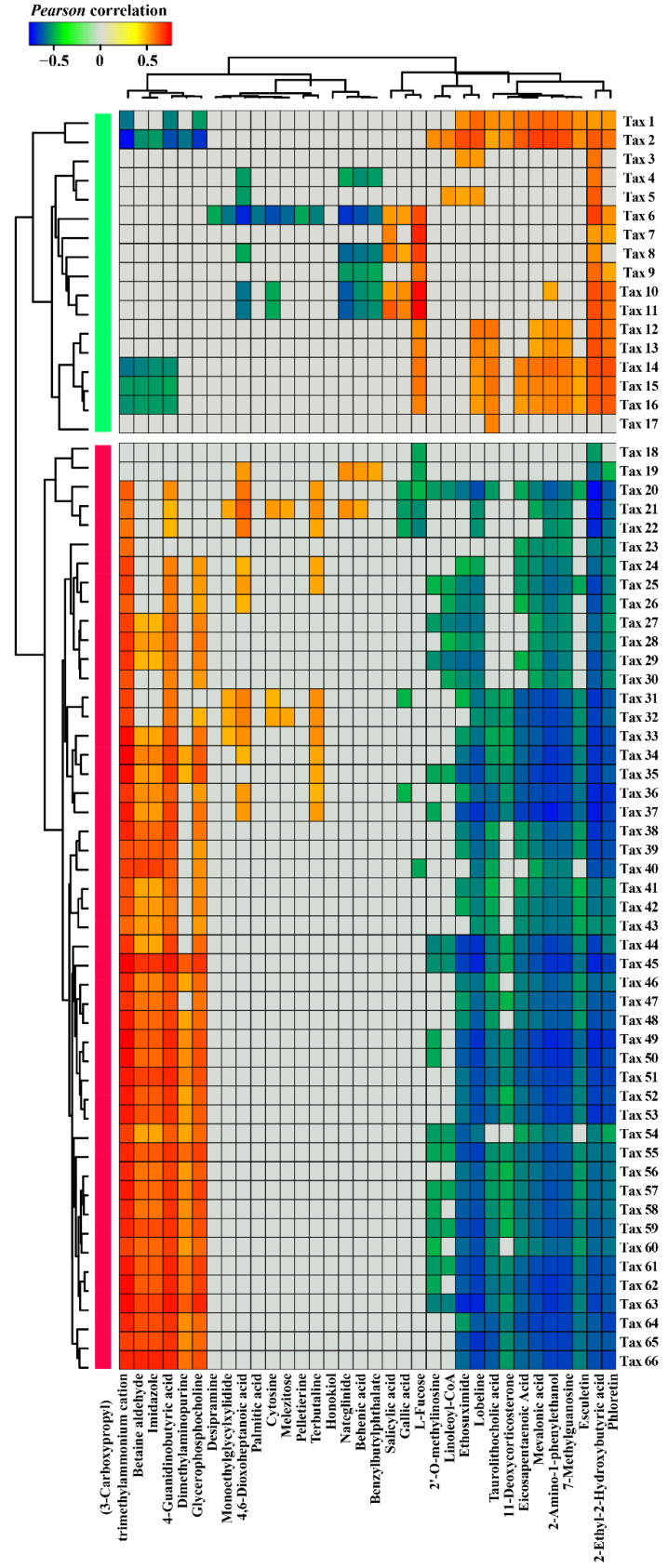
Heat map displaying correlations between differentially abundant bacterial taxa in the rhizosphere of WT and *Nr* tomato lines and root exudate metabolites. The plot displays the average Pearson correlations values between specific *Nr*-suppressed and *Nr*-enriched bacterial taxa (based on taxa relative abundances) and root exudate metabolites at the three distinct growth stages. Only *Pearson* correlation coefficients above 0.5 or below −0.5 are shown. The light green and red columns represent the 17 *Nr*-suppressed and the 49 *Nr*-enriched bacterial taxa, respectively.

We identified 17 *Nr*-suppressed bacterial taxa (based on bacterial relative abundance data) that were significantly depleted in *Nr* plants compared to WT at all three different growth stages. These 17 *Nr*-suppressed bacterial taxa showed positive correlations with 16 metabolites (type *i*) and negative correlations with 18 metabolites (type *ii*) (Figure 4). For example, significant decreases in salicylic acid, gallic acid, and L-fucose were positively associated with the lower relative abundances of *Dyadobacter* sp. (Tax 11), *Bacillus* sp. (Tax 8), and *Spirosoma* sp. (Tax 6) in *Nr* plants compared to WT (Figure 4 and Supplementary File S3). The higher concentration of betaine aldehyde in the root exudates of *Nr* plants was negatively correlated with lower abundances of *Beggiatoa* sp. (Tax 2), *Chryseolinea* sp. (Tax 14 and Tax 15), and *Ohtaekwangia* sp. (Tax 16) in the rhizosphere of *Nr* plants (Figure 4 and Supplementary File S3).

We also identified 49 *Nr*-enriched bacterial taxa (based on bacterial relative abundance data) that were significantly higher in *Nr* plants compared to WT at all three different growth stages. These taxa showed positive correlations with 18 metabolites (type *iii*) and negative correlations with 16 metabolites (type *iv*) (Figure 4). Lower concentrations of eicosapentaenoic acid and esculetin in the root exudates of *Nr* plants were negatively correlated with the higher relative abundances of *Lysobacter* sp. (Tax 52, Tax 61, Tax 65, Tax 58, Tax 48, Tax 33, Tax 47, Tax 42, Tax 55, Tax 41, Tax 46, Tax 56 and Tax 44) in the rhizosphere soil of *Nr* plants (Figure 4 and Supplementary File S3). In addition, a significant decrease in gallic acid was negatively correlated with increases in the relative abundances of *Xanthomonas* sp. (Tax 36), *Pseudoxanthomonas* sp. (Tax 31), *Pseudomonas* sp. (Tax 22 and Tax 21), and *Mizugakiibacter* sp. (Tax 20) in *Nr* plants relative to WT (Figure 4 and Supplementary File S3). A significant decrease in L-fucose was negatively correlated with significantly higher relative abundances of *Pseudomonas* sp. (Tax 22 and Tax 21), *Mizugakiibacter* sp. (Tax 20), *Luteibacter* sp. (Tax 19 and Tax 40), and *Arenimonas* sp. (Tax 18) in *Nr* plants compared to WT (Figure 4 and Supplementary File S3). Besides, the higher concentration of betaine aldehyde in the root exudates of *Nr* plants was positively correlated with significantly higher relative abundances of *Phenylobacterium* sp. (Tax 66, Tax 53, and Tax 51), *Lysobacter* sp. (Tax 52, Tax 61, Tax 65, Tax 58, Tax 48, Tax 33, Tax 47, Tax 42, Tax 55, Tax 41, Tax 46, Tax 56 and Tax 44), *Frateuria* sp. (Tax 63), *Luteimonas* sp. (Tax 60, Tax 62, Tax 35, Tax 34 and Tax 40), *Thermomonas* sp. (Tax 59 and Tax 45), *Stenotrophomonas* sp. (Tax 50), *Xanthomonas* sp. (Tax 36 and Tax 39), and *Dyella* sp. (Tax 37) in the rhizosphere of *Nr* plants (Figure 4 and Supplementary File S3).

## 4. Discussion

Recent studies have shown plant hormones (e.g., SA, JA, and ET) to exert significant influence on the assembly of the rhizosphere microbiome [19,20,50]. In this study, we show that tomato *Nr* mutant lines have significantly different taxonomic and functional profiles of the rhizosphere bacterial community compared to WT. Most interestingly, we show that some of these differences are tightly correlated with changes in root exudation metabolites between WT and *Nr* lines. This suggests that ET regulated by *NR* can actively exert an influence on the structure and function of the rhizosphere bacterial communities of tomato plants via trackable changes in root exudates.

Plant genotype is thought to exert an influence on structuring the microbiome composition and functionality [10,51,52]. Interestingly, most of the studies have been focusing on rhizosphere bacterial communities, and relatively few reports simultaneously addressed bacterial and fungal communities in the plant rhizosphere across gradients and conditions [53,54]. Here, we found a mutation in the ET reception NR in tomato to significantly alter the rhizosphere bacterial community, albeit no significant difference was detected in the rhizosphere fungal communities. This finding is consistent with others showing that ET is involved in structuring the rhizosphere bacterial communities [20]. Similarly, it was previously shown that three *bx* (Benzoxazinoids) mutants displaying a disruption in different steps of the BX pathway had larger effects on the rhizosphere bacterial communities than on the fungal-associated communities [15].

Several studies have reported distinct mechanisms by which plant hormones can modulate changes in the rhizosphere microbiomes [9,18,55]. For instance, salicylic acid was shown to influence the root microbiome via the selection of specific bacterial families during the colonization of *Arabidopsis* roots [50]. To date, no study has yet explored the impacts of ET using *Nr* tomato plants on rhizosphere microbial communities, and whether the potential changes would be mediated by differences in exudation profiles. This strategy provides the needed information to unravel potential mechanisms underpinning changes in root-microbiome selection. Addressing this requires an integrated approach that combines metagenomic sequencing with untargeted metabolomic analysis. In this sense, our study represents a holistic assessment of how the ET signaling pathway affects the root exudate metabolome and the associated rhizosphere microbiome. An interesting outcome of the root exudation metabolome analysis revealed that mutation in the gene *NR* caused major changes in the chemical composition of root exudates. This indicates that ET acts as an endogenous regulator of root exudation metabolism, in addition to its well-known role as a signaling molecule [24,56]. Still lacking is the systematic identification of the *NR* mutation influencing plant physiology and potentially the regulation of key genes or metabolic pathways dynamically affecting root exudate chemical profile in tomato plants. For instance, in a similar study, the two genes, *myc2* and *med25*, mutations in the JA signaling pathways were shown to change the composition of root exudates [19].

There is still debate on best practices to collect and profile root exudates [57,58,59]. In fact, different methods come with advantages and limitations; and these are often selected depending on the experimental design and research goals. In our study, although we used a hydroponic system (which does not fully mimic the real environment plants experience when growing in the soil), the system provided a valid characterization of the chemical composition of exudates. This system was chosen because it is difficult to fully sterilize soils, and to fully eliminate the interference of microorganisms in solid matrixes (such as soil) is a daunting—if not an impossible—task. In fact, soil microbes can actively consume, degrade, and transform root exudate molecules in a timely and dynamic manner. Specifically, low-molecular-weight organic compounds in root exudates (e.g., sugars, organic acids) can be rapidly consumed or altered by microorganisms living in the plant rhizosphere [60]. In addition, it has been reported that exudation profiles can significantly change due to the mechanical injury of roots during sampling from the soil [61]. It is worth mentioning that transfer from a soil system to hydroponics should be carefully performed (avoiding root damage and the transfer of soil particles) and plants need time to recover from such stress [62].

Mounting evidence has been supporting the idea that root microbiomes vary with the different developmental stages of plants [16,63,64]. Consistently, our study has also shown that the microbiome structure of both WT and *Nr* plants was influenced by the different plant developmental stages. Most interestingly, and as initially hypothesized, our results confirmed that such differences between the two genotypes intensified towards the later stages of plant development. One plausible explanation for this is that NR acts as a crucial regulator of ET perception, which results in differences in ET sensibility between the two lines [22,26]. In addition, as ET mediated by *NR* has a crucial role in the late stages of tomato growth (fruit ripening stage), this would explain why these differences are intensified at the later stages of plant development. Second, increasing variation in root exudation chemistry may lead to different temporal dynamics of root microbiome assembly. This nicely connects the differences in microhabitat at the root surface with the intrinsic interactions of microbial taxa [17]. In our study, we provide some pieces of evidence to support this argument, for instance, we found the reduction of gallic acid to correlate with changes in the relative abundances of several bacterial taxa, including *Xanthomonas* sp., *Pseudoxanthomonas* sp., and *Pseudomonas* sp. Using metagenome sequencing, our study integrated patterns of rhizosphere community assembly with the functional potential of these communities. The results revealed NR mutation in tomato to significantly affect the functional profiles of the rhizosphere bacterial communities, and these were linked with shifts in the relative abundance of genes within key metabolic pathways. Similarly, it was previously shown that the natural variation of *NRT1.1B* resulted in significant differences in the genetic potential of microbial communities in the rhizosphere of rice [10]. Since the functional profiles of these microbiomes were studied based on DNA, caution is warranted in terms of directly linking their taxonomic and functional composition with functionality in the system. Thus, this should be interpreted in terms of genomic potential rather than functional outcomes.

Several studies have already demonstrated that plant genotypes exert dynamic influences on the rhizosphere bacterial communities via changes in root exudation [65,66]. Here, we observed that ET regulated by *NR* modulates root exudation profiles that influence the rhizosphere bacterial communities. We also found that *Nr* plants released lower amounts of L-fucose, relative to the WT. L-fucose has been reported to be a chemoattractant to the plant growth-promoting rhizobacterium (PGPR) *Bacillus velezensis* SQR9 [67,68]. In addition, another study has revealed that L-fucose can be actively metabolized by *Campylobacter jejuni* strains [69]. Microbial substrate preferences for root exudate metabolites, such as amino acid and organic acid, can thus actively assist the selection of specific microbial taxa [17]. This fact can be linked with *Bacillus* sp. (Tax 8) being less abundant in the rhizosphere of *Nr* plants as a result of chemotactic responses towards L-fucose and/or the differences in the metabolic potential associated with the utilization of L-fucose as an energy source. In *Nr* plants, esculetin belonging to one member of coumarins showed a greater decrease in concentration. Esculetin was shown to have antibacterial activity against *Ralstonia solanacearum* [70]. Thus, it is likely that esculetin can contribute to *Ralstonia* disease suppression. In addition, coumarins have been shown to contribute to differences in microbial populations in the rhizosphere of *Arabidopsis thaliana* via antimicrobial effects [65,66]. Compared with WT, a decrease in the abundance of coumarin in *Nr* plants may explain the fact that thirteen *Lysobacters* sp. strains (Tax 52, Tax 61, Tax 65, Tax 58, Tax 48, Tax 33, Tax 47, Tax 42, Tax 55, Tax 41, Tax 46, Tax 56 and Tax 44) had higher relative abundances in the rhizosphere of *Nr* plants. Moreover, we provide several pieces of correlation-based evidence to illustrate how differences in rhizosphere bacterial communities relate to chemical differences in root exudation profiles. We advocate that further experiments are required to properly verify the potential causal effects of these specific root exudate molecules on positively or negatively affecting bacterial taxa. Moreover, this can have a direct influence on plant performance, in particular, by enhancing plant growth and defense against abiotic and biotic stresses.

Low-molecular-weight organic compounds in root exudates (e.g., amino acid) can be rapidly consumed or metabolized by microorganisms living in the plant rhizosphere [60]. We found that the bacterial communities in *Nr* plants had a significantly higher abundance of amino acid metabolism through the whole growth stage. In addition, the higher relative abundances of amino acids, such as D/L-arginine, L-alanine, and L-proline were detected in root exudates of *Nr* plants. Last, we also found the functions related to the metabolism of terpenoids and polyketides of *Nr* plants to be significantly higher in relative abundance when compared to WT. It is worth mentioning that it was reported that terpenoids and polyketides play crucial roles in phytopathogen suppression [30,52]. Thus, the recruitment of specific bacterial taxa in the rhizosphere of *Nr* plants by a such mechanism may be important for improving plant health.

Several studies in the literature have found specific correlations between microbial taxa and root metabolites based on the integration of amplicon sequencing, metagenomics, and metabolomics analyses based on in vitro experiments [17]. It is clear that our findings reported here will require a follow-up toward the mechanistic validation that integrates shifts in roots exudation (molecules and/or signals) with the differential recruitment of taxa [71]. Linking these differential molecules with plant metabolic pathways would allow us to better understand how plants can shift metabolisms towards the recruitments of potentially beneficial microbial taxa in the rhizosphere. Last, it remains still unclear the extent to which the ET receptor affects the root physiology with a direct impact on root architecture and anatomical traits [72].

Last, it is important to highlight that our overall findings and quantified effects were detectable without the activation of the ET pathway, which can occur when plants are exposed to specific stress conditions. As such, this suggests that all observed differences were detected at basal levels of ET signaling. In this case, greater differences may be expected and probably detected when the ET signaling pathway is activated in the plant, for instance, when plants are exposed to harsh environmental conditions or pathogen infection.

## 5. Conclusions

In this study, our results suggest that ET that is regulated by *NR* in tomato plants indirectly modulates the rhizosphere bacterial community via changes in root exudation. These findings provide a framework for the further development of strategies aiming at engineering beneficial plant microbiomes via changes in plant genetics and physiology. By better understanding the causality underpinning the correlations between root exudates and the structure and functions of the rhizosphere microbiome, we will potentially be able to better inform strategies for prospective designs and manipulate the structure and functioning of plant-associated microbiomes. Together, this will lead to advances in how plant genotypes, the abiotic environment, and specific conditions dynamically shift the taxonomic and functional profiles of root-associated microbiomes via trackable changes in root exudate chemistry.

## Data Availability

The metagenomic datasets of the paper is deposited in NCBI under accession number PRJNA702048.

## Supplementary Materials

The following are available online at https://www.mdpi.com/article/10.3390/microorganisms9122456/s1, Figure S1: Image of the rhizobox system used in this study (Photo credit: Yian Gu and Ruixin Fu, Nanjing Agricultural University). Figure S2: Effect of the gene *NR* on root exudate profiles. (a) Principal component analysis (PCA) of root exudate composition between WT and *Nr* plants at seedling, flowering and fruiting stages, respectively. The distribution of some metabolic classes in root exudation of WT and *Nr* plants at seedling (b), flowering (c) and fruiting (d) stages, respectively. Two-sided Welch’s *t*-test (upward and downward arrows for significantly increased and reduced abundances, respectively), * indicates *p* < 0.05, ** indicates *p* < 0.01 and *** indicates *p* < 0.001. Figure S3: Based on bacterial and fungal abundance tables at the species level of metagenomic sequencing by NCBI nr database annotation, alpha diversity of rhizosphere microbial community between WT and *Nr* plants. Shannon index (a,b) and species richness (c,d) of the rhizosphere bacterial and fungal communities of WT and *Nr* at seedling, flowering and fruiting stages, respectively. Two-sided Student’s *t*-test, *N.S* indicates no significance. Figure S4: Fungal community composition in the rhizosphere samples of WT and *Nr* plants based on relative abundance data at the species level. PCA (Principal component analysis) of the rhizosphere fungal communities in WT and *Nr* plants at the seedling (a), flowering (b) and fruiting stages (c). Figure S5: The number of shared and unique bacterial taxa between WT and *Nr* at the seedling (a), flowering (b) and fruiting stage (c), respectively. Figure S6: The functional potential of fungal communities in the rhizosphere of WT and *Nr* plants based on KEGG annotation. Principal component analysis (PCA) based on functional annotations of the rhizosphere fungal community of WT and *Nr* plants at the seedling (a), flowering (b), and fruiting (c) stages. Table S1: Total number of raw and clean reads after quality control, reads mapping ORFs, NCBI nr taxa counts and KEGG annotations based on metagenomic data of WT and *Nr* samples. Table S2: PERMANOVA table expressing the effect of plant stage (the seedling, flowering and fruiting stages), genotype (*NR*) and their interaction (stage × *NR*) on root exudation profile by root exudate metabolic ions PCA values. Significant results (*p* < 0.05) are highlighted in bold. Table S3: Number and percentage of the significantly decreased, increased and un-related metabolic ions in abundance of root exudates of *Nr* plants compared to WT at the seedling, flowering and fruiting stage, respectively. Total indicates the total number of the root exudation metabolic ions. Table S4: Summary of the statistical analysis of alpha diversity (Shannon index and species richness) of the bacterial and fungal communities, based on their corresponding taxa abundance data at species level by NR database annotation by two-sided Student’s *t*-test. Table S5: PERMANOVA table expressing the effect of plant stage (the seedling, flowering and fruiting stages) on the rhizosphere bacteria taxonomic community of WT and *Nr* tomato plants, respectively, by the bacteria taxonomic PCoA values. Significant results (*p* < 0.05) are highlighted in bold. Table S6: PERMANOVA table expressing the effect of plant stage (the seedling, flowering and fruiting stages), genotype (*NR*) and their interaction (stage × *NR*) on the rhizosphere bacteria taxonomic community of tomato plants by the bacteria taxonomic PCoA values. Significant results (*p* < 0.05) are highlighted in bold. Table S7: The number and percentage of the significantly enriched, depleted and total changed bacterial taxa affiliated to Actinobacteria, Proteobacteria and other phyla in the rhizosphere soil of *Nr* plants compared with WT at the seedling, flowering and fruiting stage, respectively. Supplementary File S1: Metabolites in root exudates that differ between WT and *Nr* samples at the seedling, flowering and fruiting stages, respectively. Metabolites were measured in positive (p) or negative (n) mode. The preprocessing results generated a data matrix that consisted of the retention time (RT), massto-charge ratio (*m*/*z*) values, and peak intensity. R package *CAMERA* was used for peak annotation after XCMS data processing. In-house MS2 database was applied in metabolites identification. Values for each ion represent the median retention time (rtmed), *m*/*z* value, predicted mass of the native compound, the ppm difference to the measured *m*/*z* value (ppm), counts across all samples, and the log Fold change plus *p*-value adjusted for false discovery. Supplementary File S2: Statistical analysis of the bacterial taxa between WT and *Nr* samples by STAMP software. Only statistically significant bacterial taxa are shown at the seedling, flowering and fruiting stages, respectively. Supplementary File S3: Pearson correlations between 66 bacterial taxa (relative abundance, RA is > 0.01%), which were consistently influenced by *NR* at the three different growth stages and 34 root exudate metabolites, which were also consistently influenced by *NR* at the three different growth stages. Since root exudate metabolome and microbiome analyses are not the same sample, it was not possible to compare the same samples directly. Accordingly, datasets were paired at random 10,000 times and the average *Pearson* correlation and *p*-value were calculated between the variance values.
