# Supplementary material for: Modulation of the Tomato Rhizosphere Microbiome via Changes in Root Exudation Mediated by the Ethylene Receptor NR
Source: Microorganisms. 2021 Nov 28;9(12):2456. doi: 10.3390/microorganisms9122456 (PMC8705318; doi:10.3390/microorganisms9122456)
Supplement: Supplementary file 1 [file microorganisms-09-02456-s001.zip › Suplementary Figures and Tables-proof.pdf]

Supplementary Figures and Tables

Supplementary Figures

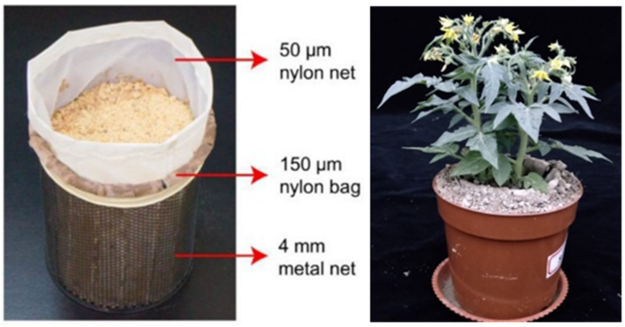

Figure S1 Image of the rhizobox system used in this study (Photo credit: Yian Gu and Ruixin Fu, Nanjing Agricultural University).

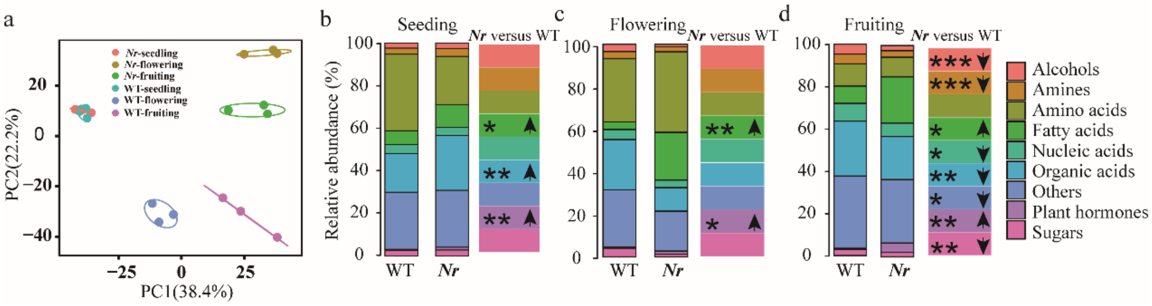

Figure S2 Effect of the gene *NR* on root exudate profiles. (a) Principal component analysis (PCA) of root exudate composition between WT and *Nr* plants at seedling, flowering and fruiting stage, respectively. The distribution of some metabolic classes in root exudation of WT and *Nr* plants at seedling (b), flowering (c) and fruiting (d) stage, respectively. Two-sided Welch's t-test (upward and downward arrows for significantly increased and reduced abundances, respectively), * indicates $p < 0.05$, ** indicates $p < 0.01$ and *** indicates $p < 0.001$.

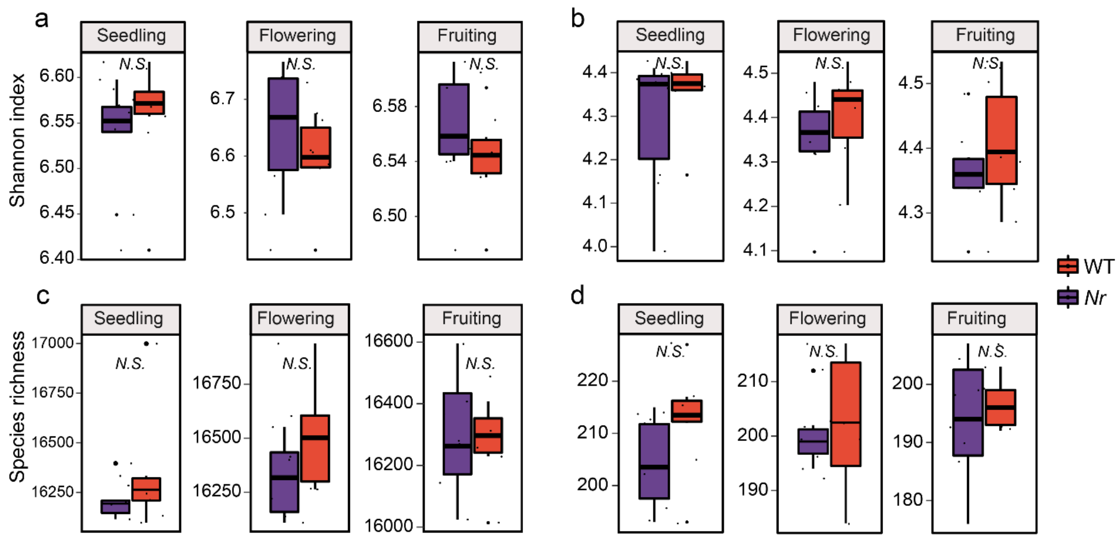

Figure S3 Based on bacterial and fungal abundance tables at the species level of metagenomic sequencing by NCBI nr database annotation, alpha diversity of rhizosphere microbial community between WT and *Nr* plants. Shannon index (a, b) and species richness (c, d) of the rhizosphere bacterial and fungal communities of WT and *Nr* at seedling, flowering and fruiting stage, respectively. Two-sided Student's t-test, *N.S* indicates no significance.

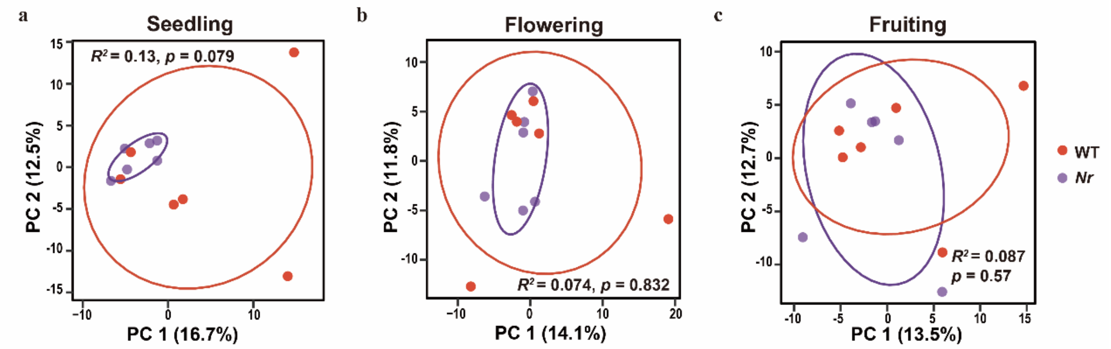

Figure S4 Fungal community composition in the rhizosphere samples of WT and *Nr* plants based on relative abundance data at the species level. PCA (Principal component analysis) of the rhizosphere fungal communities in WT and *Nr* plants at the seedling (a), flowering (b) and fruiting stages (c).

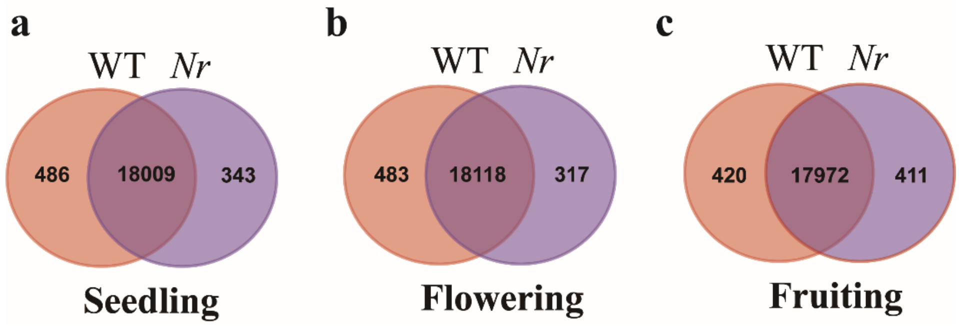

Figure S5 The number of shared and unique bacterial taxa between WT and *Nr* at the seedling (a), flowering (b) and fruiting stage (c), respectively.

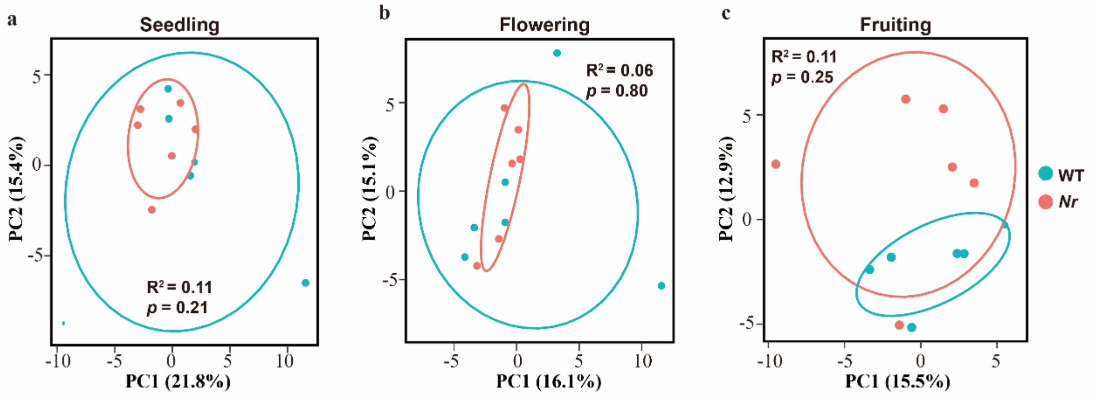

Figure S6 The functional potential of fungal communities in the rhizosphere of WT and *Nr* plants based on KEGG annotation. Principal component analysis (PCA) based on functional annotations of the rhizosphere fungal community of WT and *Nr* plants at the seedling (a), flowering (b) and fruiting (c) stages.

Supplementary Tables

Table S1 Total number of raw and clean reads after quality control, reads mapping ORFs, NCBI nr taxa counts and KEGG annotations based on metagenomic data of WT and *Nr* samples.

Sample			Raw_Reads	Clean_Reads	ORFs	Nr_Taxonomy_count	KEGG_Category_count
Nr	Seedling	1	68755666	65461016	322402	41359	48873
		2	76915304	72338606	361395	47064	56308
		3	74045610	70058284	322932	35139	44273
		4	84859092	80460250	424327	48340	57135
		5	73500236	69084492	341851	32122	39689
		6	77942304	64557858	308744	35286	45424
	Flowering	1	73205606	69716552	320769	32114	39103
		2	66675558	63069028	267784	26341	32922
		3	79918936	75346638	413284	68826	73010
		4	79305382	74682152	315090	32777	40047
		5	93554706	77185522	366707	47589	57731
		6	68819526	65584778	304906	32835	41133
	Fruiting	1	72134182	68677734	360502	33179	38606
		2	82046398	77908606	423315	45261	54361
		3	65679088	62358806	281324	26646	33630
		4	70893208	67382828	342897	37243	42277
		5	84303560	78598244	409672	47885	53613
		6	64975200	61306366	284299	28521	36425
WT	Seedling	1	73457437	64051270	196603	17783	21109
		2	74495302	69405164	303102	28740	35993
		3	68662090	64960782	316057	27952	35281
		4	68902218	64699110	298521	25353	31114
		5	76916494	72263784	365001	34982	41171
		6	69350824	65146616	321814	33036	41007
	Flowering	1	69119526	64116636	285018	23829	30546
		2	77504684	72674682	378602	40706	47385
		3	72931890	69045126	349641	39161	44401
		4	66839068	62118774	275938	27302	35726
		5	75423194	70906696	333362	37779	45481
		6	73457437	54657284	132254	11727	14628
	Fruiting	1	74186098	67210788	357354	50072	53362
		2	70989740	69439490	329987	45304	52480

3	73279534	69289142	332845	39684	44116
4	70546992	66429596	320776	34301	40568
5	78585630	74471896	336726	48206	52973
6	74952096	70801422	340923	40964	48610

Table S2 PERMANOVA table expressing the effect of plant stage (the seedling, flowering and fruiting stages), genotype (*NR*) and their interaction (stage x *NR*) on root exudation profile by root exudate metabolic ions PCA values. Significant results ($p < 0.05$) are highlighted in bold.

**Factors**	**DF**	**R^2**	**Pr (>F)**	
Stage	1	0.67963	**0.001**	***
Genotype	3	0.11293	**0.001**	***
Interaction	3	0.15085	**0.001**	***
Residuals	40	0.05659		

Table S3 Number and percentage of the significantly decreased, increased and un-related metabolic ions in abundance of root exudates of *Nr* plants compared to WT at the seedling, flowering and fruiting stage, respectively. Total indicates the total number of the root exudation metabolic ions.

**Metabolites in root exudates**	**Seedling**		**Flowering**		**Fruiting**	
	**Number**	**Percent (%)**	**Number**	**Percent (%)**	**Number**	**Percent (%)**
Decreased	501	18.51	365	13.48	361	13.34
Increased	225	8.31	1222	45.14	494	18.25
No significance	1981	73.18	1120	41.37	1852	68.42
Total	2707	100	2707	100	2707	100

Table S4 Summary of the statistical analysis of alpha diversity (shannon index and species richness) of the bacterial and fungal communities, based on their corresponding taxa abundance data at species level by NR database annotation by two-sided Student's *t*-test.

	**Alpha diversity**	**Group (WT vs *Nr*)**	**t**	***p* value**
Based on bacterial abundance data at species level	Shannon index	Seedling	-0.254	0.805
		Flowering	1.04	0.324
		Fruiting	1.42	0.187
	Species richness	Seedling	-1.10	0.298
		Flowering	-1.56	0.150
		Fruiting	0.27	0.793
Based on fungal abundance data at species level	Shannon index	Seedling	-0.846	0.417
		Flowering	-0.825	0.429
		Fruiting	-0.911	0.384
	Species richness	Seedling	-1.46	0.176
		Flowering	-0.362	0.725
		Fruiting	-0.558	0.589

Table S5 PERMANOVA table expressing the effect of plant stage (the seedling, flowering and fruiting stages) on the rhizosphere bacteria taxonomic community of WT and *Nr* tomato plants, respectively, by the bacteria taxonomic PCoA values. Significant results ($p < 0.05$) are highlighted in bold.

**Factors**	**Df**	R^2	**Pr(>F)**	
WT _flowering vs WT _seedling	1	0.18805	**0.026**	*
Residuals	10	0.81195		
WT _fruiting vs WT _flowering	1	0.22427	**0.002**	**
Residuals	10	0.77573		
*Nr*_ flowering vs *Nr* seedling	1	0.22758	**0.024**	*
Residuals	10	0.77242		
*Nr*_ fruiting vs *Nr*_ flowering	1	0.185	**0.036**	*
Residuals	10	0.815		

Table S6 PERMANOVA table expressing the effect of plant stage (the seedling, flowering and fruiting stages), genotype (*NR*) and their interaction (stage x *NR*) on the rhizosphere bacteria taxonomic community of tomato plants by the bacteria taxonomic PCoA values. Significant results ($p < 0.05$) are highlighted in bold.

**Factors**	**Df**	**R^2**	**Pr (>F)**	
Stage	2	0.22504	**0.001**	***
Genotype	1	0.06769	**0.002**	**
Interaction	2	0.06999	**0.036**	*
Residuals	30	0.63728		

Table S7 The number and percentage of the significantly enriched, depleted and total changed bacterial taxa affiliated to Actinobacteria, Proteobacteria and other phyla in the rhizosphere soil of *Nr* plants compared with WT at the seedling, flowering and fruiting stage, respectively.

**Stage**	**Phyla**	**Enriched**		**Depleted**	
		**Number**	**Percent (%)**	**Number**	**Percent (%)**
Seedling	Proteobacteria	459	2.44	185	0.98
	Actinobacteria	163	0.87	140	0.74
	Others	70	0.37	782	4.15
	Total	692	3.67	1107	5.88
Flowering	Proteobacteria	225	1.19	439	2.32
	Actinobacteria	435	2.30	24	0.13
	Others	115	0.61	688	3.64
	Total	775	4.10	1151	6.08
Fruiting	Proteobacteria	501	2.66	168	0.89
	Actinobacteria	96	0.51	101	0.54
	Others	292	1.55	526	2.80
	Total	889	4.73	795	4.23

Note: The total number of the bacterial taxa at the three different stages was 18838, 18918 and 18803, respectively.
